# GABB: A global dataset of alpine breeding birds and their ecological traits

**DOI:** 10.1038/s41597-022-01723-6

**Published:** 2022-10-15

**Authors:** Devin R. de Zwaan, Davide Scridel, Tomás A. Altamirano, Pranav Gokhale, R. Suresh Kumar, Steven Sevillano-Ríos, Arnaud G. Barras, Libertad Arredondo-Amezcua, Addisu Asefa, Ricardo A. Carrillo, Ken Green, Carlos A. Gutiérrez-Chávez, Aleksi Lehikoinen, Shaobin Li, Ruey-Shing Lin, Christopher J. Norment, Krista N. Oswald, Alexey A. Romanov, Julio Salvador, Kerry A. Weston, Kathy Martin

**Affiliations:** 1 Department of Forest and Conservation Sciences, University of British Columbia, Vancouver, British Columbia Canada; 2 Centro Nazionale delle Ricerche, Istituto di Ricerca sulle Acque, Brugherio, Monza e della Brianza Italy; 3 Audubon Americas, National Audubon Society, Santiago, Región Metropolitana Chile; 4 Cape Horn International Center for Global Change Studies and Bicultural Conservation, Universidad de Magallanes, Punta Arenas, Región de Magallanes Chile; 5 Department of Endangered Species Management, Wildlife Institute of India, Dehradun, Uttarakhand India; 6 Department of Natural Resources, Cornell Lab of Ornithology, Cornell University, Ithaca, New York USA; 7 Centro de Ornitologia y Biodiversidad (CORBIDI), Santiago de Surco, Lima Peru; 8 Division of Conservation Biology, Institute of Ecology and Evolution, University of Bern, Bern, Canton of Bern Switzerland; 9 Swiss Ornithological Institute, Sempach, Lucerne, Switzerland; 10 Independent researcher, Morelia, Michoacán Mexico; 11 Department of Conservation Ecology, Faculty of Biology, Philipps-University of Marburg, Marburg, Hessen Germany; 12 Ethiopian Wildlife Conservation Authority, Addis Ababa, Oromia Ethiopia; 13 College of Asia and the Pacific, Australian National University, Canberra, Australian Capital Territory Australia; 14 Helsinki Lab of Ornithology, Finnish Museum of Natural History, University of Helsinki, Helsinki, Uusimaa Finland; 15 Department of Zoology, College of Life Sciences, Yangtze University, Jingzhou, Hubei China; 16 Habitats and Ecosystems Division, Endemic Species Research Institute, Jiji, Nantou Taiwan; 17 Department of Environmental Science and Ecology, State University of New York College at Brockport, Brockport, New York USA; 18 Mitrani Department of Desert Ecology, Jacob Blaustein Institute of Desert Research, Ben-Gurion University of the Negev, Be’er-Sheva, Israel; 19 Department of Zoology and Entomology, Rhodes University, Mkhanda, Eastern Cape South Africa; 20 Department of Biogeography, Faculty of Geography, Lomonosov Moscow State University, Moscow, Podmoskovye Russia; 21 BirdsRussia, Moscow, Podmoskovye Russia; 22 Biodiversity Group, Department of Conservation, New Zealand Government, Christchurch, Canterbury New Zealand; 23 Pacific Wildlife Research Centre, Environment and Climate Change Canada, Vancouver, British Columbia Canada

**Keywords:** Biodiversity, Biogeography, Conservation biology, Community ecology

## Abstract

Alpine ecosystems represent varied climates and vegetation structures globally, with the potential to support rich and functionally diverse avian communities. High mountain habitats and species are under significant threat from climate change and other anthropogenic factors. Yet, no global database of alpine birds exists, with most mountain systems lacking basic information on species breeding in alpine habitats, their status and trends, or potential cryptic diversity (i.e., sub-species distributions). To address these critical knowledge gaps, we combined published literature, regional monitoring schemes, and expert knowledge from often inaccessible, data-deficient mountain ranges to develop a global list of alpine breeding bird species with their associated distributions and select ecological traits. This dataset compiles alpine breeding records for 1,310 birds, representing 12.0% of extant species and covering all major mountain regions across each continent, excluding Antarctica. The Global Alpine Breeding Bird dataset (GABB) is an essential resource for research on the ecological and evolutionary factors shaping alpine communities, as well as documenting the value of these high elevation, climate-sensitive habitats for conserving biodiversity.

## Abstract (summary)

**Table:** 

Measurement(s)	Breeding specialization • Breeding status • Migration behaviour • Nest type • Nest site • Data reliability • Endemism • IUCN status
Technology Type(s)	Literature review, field monitoring, expert knowledge • Literature review, expert knowledge • Literature review • IUCN red list
Sample Characteristic - Organism	Aves
Sample Characteristic - Environment	alpine • nival

## Background & Summary

Mountains represent biodiversity hotspots, with elevational gradients promoting rapid turnover of habitats, climate, and subsequently, faunal communities^1,2^. As globally significant landscape features, mountains comprise ~25% of the total landmass, yet support a disproportionate amount of biodiversity, including over 50% of regional species richness in some regions^3–5^. High mountain habitats are often in remote, difficult to access locations, such that systematic breeding or population surveys are rare, leading to critical data gaps in our understanding of avian communities and basic life-history traits^6,7^. This is especially true for alpine and nival ecosystems (the habitats above climatic treeline), which are particularly susceptible to the effects of climate change^8^. A critical need for mountain bird research and conservation is to have baseline data on specific avian communities, such as those in alpine ecosystems.

Alpine or above treeline habitats occur on every continent and make up about 2.6% of the global landmass, excluding Antarctica^9,10^. These habitats experience environmental extremes (e.g., low temperatures, high wind speeds, heavy winter precipitation), which limit upright tree growth and result in open habitats of cold-adapted grasses, shrubs, or stunted tree patches^11^. Alpine habitats are generally perceived as being relatively devoid of life, with avian diversity often dropping sharply above treeline compared to the montane forest community below treeline^12^. However, at a global scale, alpine habitats exhibit remarkable variation, from high latitude tundra to the Andean *Puna* grasslands and *Polylepis* tree patches, the Trans-Himalayan alpine deserts, or the ericoid scrub of the Afroalpine^13–15^. These differences promote niche diversity for a wide variety of bird species, leading to alpine bird communities that are distinct from both lower elevation communities, as well as nearby mountain ranges (i.e., high endemism^16,17^). Yet, despite their unique contribution to mountain diversity and potential climate sensitivity as cold-adapted organisms, alpine bird communities are among the least studied avian systems, limiting our understanding of their differentiation among regions, species distributions, or life-history^7^.

To provide a baseline for future research, we assembled a global list of birds known to breed in alpine habitats with information on their distribution across mountain ranges and region-specific species traits. Several past attempts have been made to classify and compare high mountain bird communities at different spatial scales. Dorst and Vuilleumier^18^ first compared alpine birds between the tropical Andes, Africa, and temperate Tibet using field observations. Subsequent efforts assembled lists of mountain birds globally^19^, across the Holarctic^7^, or continentally^20^, using BirdLife International range maps or ringing station data. However, these datasets are either limited in scale (i.e., continental) or identify species by intersecting range maps with mountain boundaries at a relatively coarse level, such that they do not differentiate between the below and above treeline community (i.e., montane forest and treeless alpine habitats). These approaches also do not contain information about breeding activity, only presence, which could include post-breeding use and seasonal movements. Using field observations validated by expert knowledge across all continents, we provide the first global list of alpine breeding birds at a scale that facilitates combinations with increasingly accessible climate, habitat, and avian trait databases.

**Fig. 1:**
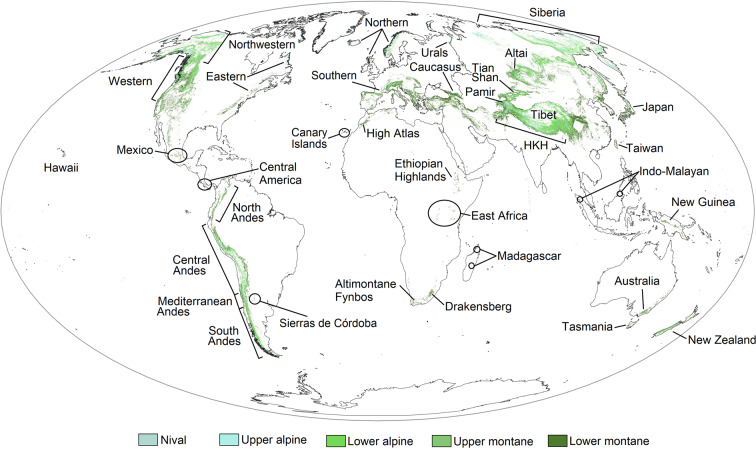
Location of the main mountain regions included in the dataset, with labels corresponding to descriptions in Tables 2 and 3. Colours denote different thermal belts following Korner *et al*.^9^, with our definition of alpine habitat corresponding approximately with nival, upper alpine, lower alpine, and upper montane classifications. Not all subdivisions of the Hindu Kush-Himalayan Arc (HKH) and Tibet regions are included due to space limitations. See Tables 2 and 3 for full descriptions of each region. The raw data used to create the map originated from the Global Mountain Biodiversity Project^27,28^ and were downloaded from the U.S. Geological Survey website (https://rmgsc.cr.usgs.gov/gme/)^36^.

The Global Alpine Breeding Bird dataset (GABB) is unique in that it provides alpine habitat-specific data on avian breeding status in each major mountain range globally (Fig. 1). Multiple avian trait databases have been assembled recently, covering most extant species, including those that inhabit mountain regions. These databases vary by the species traits they present, but together provide extraordinary resources for broad-scale analyses of bird communities. The GABB dataset fills several important knowledge gaps, facilitating combinations with these existing avian databases (Table 1). GABB represents the first database to distinguish between birds that breed above treeline within major mountain regions versus general presence based on coarse species range maps. It also incudes nest trait data which characterizes the alpine nesting niche of each species. While certain traits, such as migration behaviour, are included in other global databases (e.g., AVONET^21^; Table 1), we provide traits specific to alpine breeding populations which may differ from the predominant global trait. For example, low elevation populations of certain species may be year-round residents, while alpine-breeding populations may conduct altitudinal or short-distance latitudinal migration. These finer-scale details are required to evaluate the current and future threats to alpine breeding birds, as well as the fundamental characteristics of bird communities that are adapted to breeding above treeline.

**Table 1:** Comparison of the Global Alpine Breeding Bird dataset (GABB) to other existing global-scale trait databases.

	GABB	Tobias *et al*. (2022)	Sheard *et al*. (2020)	Bird *et al*. (2020)	Scholer *et al*. (2020)	Jetz *et al*. (2018)	Wilman *et al*. (2014)	Jetz *et al*. (2012)	La Sorte & Jetz (2010)
Alpine breeding	X								
High elevation	X								X
Migration	X	X							
Nest traits	X								
Endemism	X								
Conservation status	X								
Range size		X				X			X
Elevational range		X				X			
Dietary guild		X					X		
Foraging niche		X					X		
Body size		X					X		
Wing shape		X	X						
Generation time				X					
Annual survival				X	X				
Phylogenetic distance								X	

The GABB dataset occupies novel space in the literature with respect to alpine breeding status, ecological traits of alpine breeding populations, and traits relevant to the conservation of high elevation species. The dataset is formatted to facilitate combination with other databases, such as those represented here, to address broad-scale ecological and evolutionary questions for high mountain birds. Migration behaviour in GABB is specific to alpine breeding populations compared to Tobias *et al*.^21^, which is generalized across elevations. See text and metadata for more details.

In addition to global trait values, certain species traits can differ among subspecies and populations. Thus, we also include region-specific traits for each species to provide the data necessary to address fundamental questions in ecology, evolution, and biogeography at a global scale. For example, alpine birds in certain mountain regions may exhibit a greater propensity for short- versus long-distance migration, and these differences can help reveal the processes that produced and continue to shape alpine bird communities. Finally, the role of high mountain habitats in promoting resilience to climate and land-use change is unclear. High mountain habitats may provide refugia for species experiencing population declines in response to habitat loss at lower elevations^22^. Beyond variables that characterize alpine breeding status and ecological traits, we also include information that is relevant to the conservation of alpine communities, including mountain endemism and global conservation status (Table 1). Thus, this dataset provides a baseline that is necessary to evaluate the biodiversity and conservation value of alpine habitats, which will be regularly updated as new information becomes available.

## Methods

### Defining alpine habitat and mountain regions

**Fig. 2:**
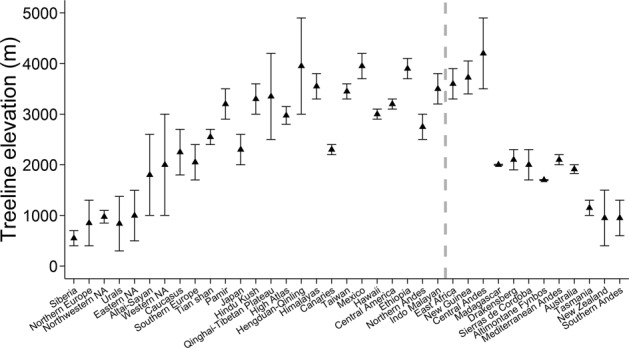
The median (triangular points) and range (error bars) of treeline elevation for each of the main mountain regions covered in the dataset (Fig. 1). The mountain regions are arranged from north to south (left to right) and the grey dashed line represents the relative position of the equator. Treeline elevation was derived from different sources depending on the region (see ‘Data sources’ in the dataset). The abbreviation ‘NA’, such as in ‘Northwestern NA’, refers to North America.

We defined alpine habitat as the area above climatic treeline, including the nival belt, where temperature, wind, drought, snow, or nightly frost limit vegetation growth to shrubs, krummholz, or fragmented tree patches less than 3 m in height^3,23,24^. Realized treeline can be markedly lower than the climatic treeline due to the absence of continuous forest at lower elevations, or human activities such as logging, burning, and livestock grazing^25^. While anthropogenically influenced treeline produces habitat reminiscent of alpine meadows, these habitats are not climatically representative of alpine ecosystems and thus they were not included when assembling this dataset. Climatic treeline elevation varies globally based on latitude, topography, aspect, and proximity to the coast (i.e., oceanic influence)^11^. Therefore, we defined alpine habitat separately for each mountain region based on local climate and published accounts of alpine vegetation. While alpine habitats usually occur above at least 1,500 m elevation globally, at high latitudes (>55°N or 41°S) this elevation can be as low as ~400 m^26^ (Fig. 2).

The alpine habitats we identified broadly align with the ‘lower alpine’, ‘upper alpine’, and ‘nival’ belts mapped by Korner *et al*.^9^ and made available by the Global Mountain Biodiversity Assessment project (http://www.mountainbiodiversity.org/explore)^27,28^. However, certain areas, such as the Sierras de Córdoba, Argentina or the Isthmian Páramo on volcanoes in Central America were classified as ‘upper montane’ by Korner *et al*.^9^ based on thermal belts alone. For the purposes of this dataset, we considered these regions alpine habitat based on published measurements of treeline and distinct alpine plant communities facilitated by a mixture of temperature, precipitation, nightly frost, and wind constraints. For example, the Drakensberg range in South Africa was identified as ‘upper montane’ only, but botanical studies have characterized the region as *Themeda-Festuca* grassland from 1,900–2,800 m and alpine heathlands above 2,800 m^13^, representing extensive habitat above treeline. As a result, our definition of alpine habitat expands on the thermal belts mapped by Korner *et al*.^9^. In this way, the avian communities we identified retain species lineages that are confined to cooler high elevation habitats, representing remnants of more extensive alpine ecosystems from the last glaciation event.

We grouped mountain ranges into 12 global regions and 38 subregions based on similar climates and alpine vegetation stemming from shared geographic position (Tables 2, 3; Fig. 1). The ‘Islands’ category represents very limited alpine habitat on four disparate islands that do not easily fit within any other major region, but nevertheless occur in subtropical or tropical realms: Hawaii, Sumatra, Borneo, and the Canary Islands. Alpine breeding birds and life-history traits were identified for each individual region so that future analyses can either include or remove mountain ranges depending on their definition of alpine habitat. This approach also promotes comparisons of avian communities at a finer scale across the full diversity of alpine habitats.

**Table 2:** Description of the major regions and specific mountain ranges in the Americas that are included in the dataset.

Mountain region	Description
**North America**	
*Northwestern ranges*	Northern British Columbia, Yukon Territory, and Alaska up to the Brooks Range at ~68°N. Includes alpine-Arctic transitional tundra between 400 and 1,000 m above sea level
*Western ranges*	Rocky Mountains, Coast Mountain Range, Cascade Mountains, and Sierra Nevada.
*Eastern ranges*	Acadian-Appalachian range and the Labrador highlands
**Mesoamerica**	
*Mexico*	Faja Volcánica Transmexicana (Trans-Mexican Volcanic belt)
*Central America*	Isthmian Páramo in Costa Rica and Panama
**Tropical South America**	
*Northern Andes*	Páramo; tropical wet Andes; Venezuela, Colombia, and Ecuador (~9°N to 4°S)
*Central Andes*	*Puna* and *Polylepis* patches; tropical dry Andes; Peru, Bolivia, northern Chile, and northwest Argentina (~4°S to 30°S)
**Temperate South America**	
*Mediterranean Andes*	~30°S to 35°S; high Andes in Chile and Argentina
*Sierras de Córdoba*	Four isolated ranges in Córdoba, Argentina (31.5°S)
*Southern Andes*	~35°S to 56°S; includes alpine tundra above 500 m in the far south of Chile and Argentina

Each region is a column where confirmation of breeding in an alpine habitat is denoted by ‘1’. Latitudinal delineations of high Andean habitats were derived from Sevillano-Ríos *et al*.^14^.

**Table 3:** Description of the major regions and specific mountain ranges from Eurasia, Africa, and Oceania, plus the miscellaneous mountain ‘Islands’ region.

Mountain region	Description
**Europe**	
*Northern ranges*	Scandinavian mountains, Scottish Highlands, & Iceland
*Southern ranges*	Alps, Pyrenees, Apennines, Dinaric Alps, & Carpathians
*Caucasus*	Greater & Lesser Caucasus ranges & the Anatolian Highlands
*Urals*	Ural Mountains, including the high latitude Polar Urals
**Africa**	
*Atlas Mountains*	High Atlas, Morocco; above a Juniper spp. treeline
*Ethiopian Highlands*	Afroalpine habitat in Ethiopia and Eritrea
*East Africa*	Primarily Mount Kilimanjaro (Tanzania), Mount Kenya (Kenya), & the Rwenzori Mountains (Uganda)
*Drakensberg*	*Themeda-Festuca* alpine veld & Maloti-Drakensberg alpine heathlands in Lesotho (independent kingdom within South Africa).
*Altimontane Fynbos*	Cape Fold Mountains, South Africa; primarily the Swartberg Range; highest elevation fynbos habitat.
*Madagascar*	Andringitra Massif, and the Tsaratanana & Ankaratra Ranges
**North Asia**	
*Tian Shan*	Tian Shan mountain system, Kazakhstan
*Altai-Sayan*	Altai and Sayan ranges in Mongolia and southern Siberia.
*Siberia*	Putorana Plateau; Verkhoyansk, Chersky & Dzhugdzhur ranges east to the Sea of Okhotsk and Bering Sea
**Tibet**	
*Pamir ranges*	Pamir Ranges on the western boundary of the Tibetan Plateau
*Qinghai-Tibet*	The Qinghai-Tibetan Plateau
*Hengduan-Qinling*	Southeastern and eastern extensions from the Tibetan Plateau
**Hindu Kush-Himalayan Arc (HKH)**	
*Hindu Kush*	Runs along the border of Afghanistan & NW Pakistan to SE Tajikistan
*Himalayas*	Extends along an arc through Pakistan, India (Jammu & Kashmir in the west to Arunachal Pradesh in the east), Nepal, & Bhutan
*Trans-Himalayas*	Mountain range running parallel to & north of the Himalayas; consists of desert steppe and dry scrub
**East Asia**	
*Japan*	The Japanese Alps in the central Honshu province
*Taiwan*	Central Mountain & Hsüeh-shan ranges
**Australasia**	
*Australia*	Snowy Mountains in southeastern Australia
*Tasmania*	Central Highlands
*New Zealand*	Central Volcanic Plateau on the North Island, the Southern Alps & the northwest ranges on the South Island, & Stewart Island (above ~400 m)
*New Guinea*	Maoke range in Papua & the New Guinea Highlands, plus scattered mountains in Papua New Guinea
**Islands**	
*Hawaii*	Primarily Mauna Kea and Mauna Loa
*Canary Islands*	Highest elevations on Tenerife and La Palma
*Indo-Malayan*	Mount Kinabalu, Borneo & Mount Kerinci, Sumatra

Each region is a column where confirmation of breeding in an alpine habitat is denoted by ‘1’.

### Alpine breeding bird species

For each region described in Tables 2 and 3, we assembled a list of alpine breeding species from published literature, environmental assessment reports, regional monitoring schemes, bird atlases, and expert knowledge following the most recent International Ornithology Committee taxonomy, version 12.1^29^. An alpine breeding bird is any species that nests above treeline, regardless of how frequently, such that all or a certain proportion of a species is dependent on alpine habitat during the breeding season. Due to certain data-deficiencies underlying existing species range estimates above treeline, using knowledge from regional experts was the most accurate method to assemble a global list of alpine breeding birds for most mountain regions. See the Technical Validation section for specifics on how we validated the use of expert knowledge when assembling species and their traits for the Global Alpine Breeding Bird list.

### Species traits

**Fig. 3:**
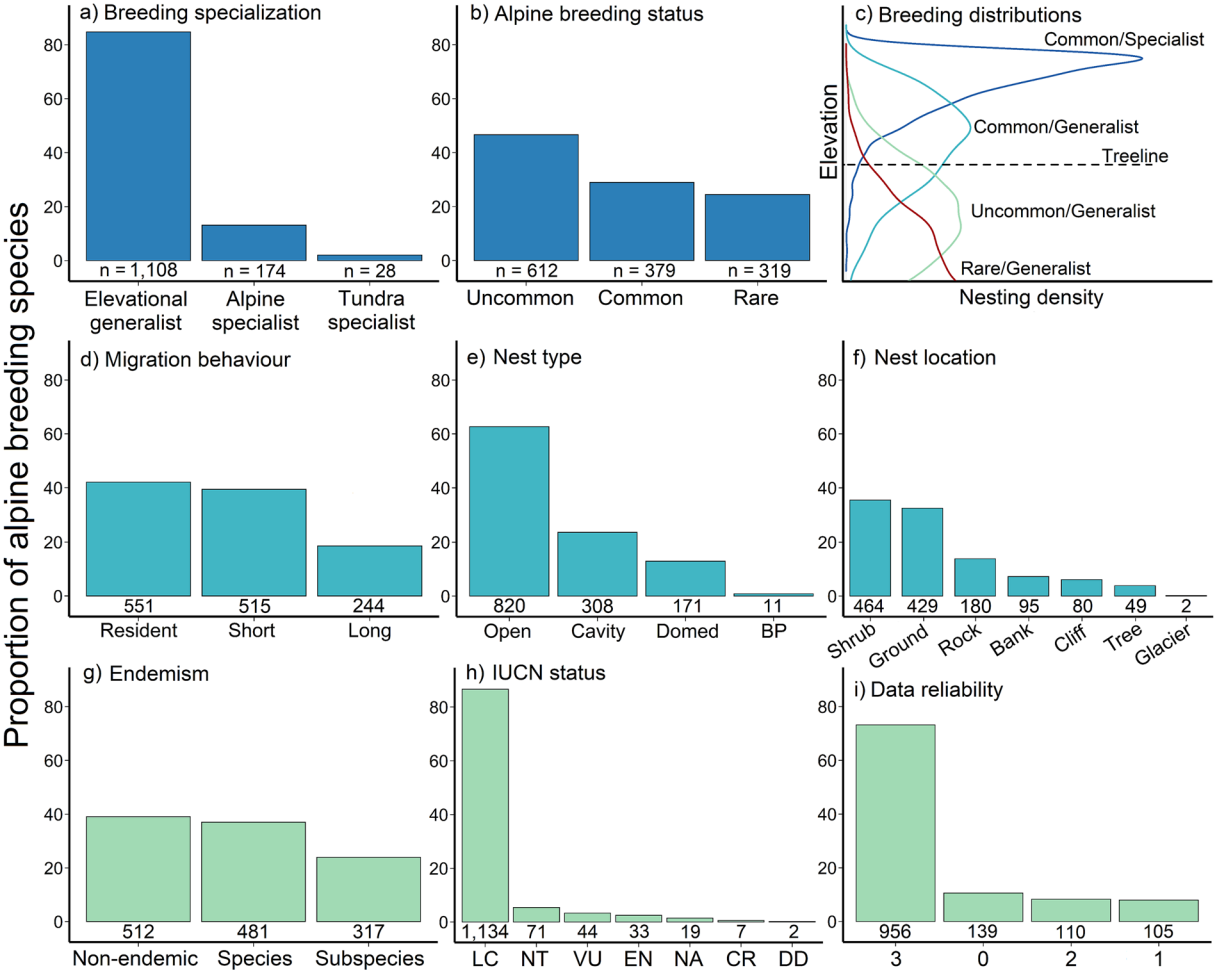
The global distribution for each trait included in the dataset, including (**a**–**c**) alpine breeding propensity, (**d**–**f**) ecological traits, and (**g**–**i**) traits relevant to conservation status and data uncertainty. In all cases except panel c the y-axis is the proportion of all 1,310 alpine breeding species identified in the dataset. Panel c depicts the elevational breeding distribution expected from the different combinations of breeding specialization and alpine breeding status to visualize the probability of breeding above treeline. In Panel e, ‘BP’ refers to brood parasite. See Table 4 or the metadata for full descriptions of each trait.

We included species traits that fall under three general topics: 1) alpine breeding propensity, 2) ecological traits, and 3) conservation value. Alpine breeding propensity includes breeding habitat specialization and alpine breeding status, ecological traits include migration behaviour and nest traits, while conservation value encompasses mountain endemism and conservation status. Together, these variables broadly reflect alpine habitat use during the breeding season globally, as well as provide the basis for evaluating the conservation potential and risks for alpine bird communities. We recorded general trait specifications for each species using available resources such as Birds of the World^30^, the IUCN Red List^31^, and AVONET^21^. We then solicited region-specific traits from regional experts and the same review process was conducted for these traits as for alpine breeding evidence (see Technical Validation). All traits were specific to alpine breeding birds whenever possible. The global distribution of each species trait can be visualized in Fig. 3.

Specialization for breeding in alpine habitats (hereafter ‘breeding specialization’) and the propensity to breed in alpine habitats (hereafter ‘breeding status’) form a tiered estimate of alpine breeding behaviour. First, we classified each species into one of three breeding specialization categories to differentiate among species that predominantly breed above treeline (alpine specialists), breed both above and below treeline (elevational generalists) or are limited to high latitude tundra habitats (tundra specialists). The latter includes alpine-Arctic or alpine-Antarctic transition zones, where species nest in higher, drier tundra (approximately >400 m elevation) but may also breed in wet tundra at lower or coastal elevations. In this way, we clearly identified species that breed in alpine tundra habitat, but where tundra is the primary driver of breeding presence, not necessarily selection for high elevation. Under breeding status, we quantified the likelihood of breeding above treeline relative to below treeline as common, uncommon, or rare. Alpine specialists are always common alpine breeders (regardless of their density and distribution), but generalists or tundra specialists can be common, uncommon, or rare breeders in alpine habitats depending on whether they are found breeding consistently above treeline, more often breeding below treeline, or only incidentally breeding in the alpine, respectively. Together, these variables identify a species’ relative probability of breeding along the elevational gradient and with respect to the treeline (Fig. 3).

We used two nest traits to identify the general breeding niche of each species: nest type and nest site. Nest type included three primary category levels (open cup, cavity, domed nest), while nest site was subdivided into seven levels (ground, bank, shrub, tree, rock, cliff, and glacier). Brood parasite species, which will use a range of nest types and sites depending on the host species, were placed in an additional ‘brood parasite’ category for each nest trait. A species with an open cup or domed nest is limited to placing the nest on the ground, in vegetation (e.g., a shrub or stunted tree), or on a cliff, while cavity nesters may be in a bank (i.e., burrow/tunnel), in a rock (e.g., crevice), or in a tree (e.g., natural or excavated cavity). If nest traits were undescribed for a certain species, we inferred nest traits from the most closely related species in similar high elevation habitats (see Data uncertainty).

Species were assigned to three migration categories based on their predominant behaviour: resident, short-distance, and long-distance migrants. Resident species remain near their breeding habitat year-round, allowing for occasional, short-term movements in response to extreme weather events. Short-distance migrants conduct seasonal altitudinal migrations, short latitudinal migrations, or nomadic movements where the species remains within the general breeding region (e.g., within the temperate zone). Long-distance migrants travel extensive distances to winter in an entirely different region than their breeding habitat (e.g., temperate breeders to tropical habitats). A general threshold of 3,000 km was used to distinguish between short- and long-distance migrants because it approximates the distance traveled from the Himalayas to the southern coast of India, Northern Europe to the Mediterranean coast, or Alaska to California. In other words, this distance represents a relatively consistent reference across global regions. While there are finer-scale migration designations that could be made, such as partial or altitudinal migration, we lack detailed movement data for most species and regions. Although a global list of potential altitudinal migrants exists that can be incorporated with this alpine breeding bird dataset if desired^32^, altitudinal migration often co-occurs with short-distance latitudinal movements and there are considerable differences in migration behaviour among subspecies, populations, and even individuals^33^. We therefore chose to use established migration categories that align with other global trait databases. In fact, our migration designations were largely congruent with those in AVONET^21^, with the primary difference being between resident and short-distance migrants. We identified ~200 short-distance migrants that were considered sedentary (resident) under the AVONET classification. This difference is to be expected given that we defined migration behaviour for alpine breeding populations compared to global trait values for all populations. For many species, alpine breeding birds will depart higher elevations during winter to avoid severe weather conditions, even though low elevation populations of the same species may be predominantly resident^34^. Therefore, the three broad categories chosen here are intended to balance available information with sufficient accuracy to provide data useful for large-scale life-history and biogeographic analyses of alpine breeding birds.

Mountain endemism refers to a species whose breeding range is restricted by physical, environmental, or biological barriers to a general mountain region and the surrounding low elevation habitat. For example, a species breeding only on the Tibetan Plateau was classified as an endemic species, but a species that breeds across the Tibetan Plateau, the Himalayas, and the Altai Mountains was classified as non-endemic. When possible, we also classified endemism for defined subspecies. Species endemism is a more conservative metric, while subspecies endemism attempts to estimate additional cryptic endemism given that species differentiation is not well-defined for many high elevation birds. For example, the Caucasus Mountains support several distinct subspecies isolated from their primary distributions, including the Great rosefinch (*Carpodacus rubicilla rubicilla*), Dunnock (*Prunella modularis obscura*), and Güldenstädt’s redstart (*Phoenicurus erythrogastrus erythrogastrus*).

Finally, conservation status refers to the IUCN Red List designations, version 2022-1^31^. In addition to the traditional IUCN categories (e.g., Least Concern, Near Threatened, Vulnerable, etc.), we also included a Not Assessed (NA) category that generally occurred when a species was recently split. See Table 4 for complete definitions of all traits.

**Table 4:** Definitions of species traits included in the Global Alpine Breeding Bird dataset.

Trait	Trait level	Definition
**Breeding specialization**		**Predominant breeding habitat**
	*Alpine*	Alpine specialist; primarily breeds above treeline
	*General*	Elevational generalist; breeds both above and below treeline
	*Tundra*	Breeds only in high latitude alpine and polar tundra; occurs at alpine-Arctic transition zones where species are selecting for tundra habitat
**Breeding status**		**Breeding propensity in alpine relative to other habitats**
	*Common (C)*	Nests in alpine across geographic range; equal to or more likely to nest in the alpine than in lower elevation habitats
	*Uncommon (U)*	Often breeds in alpine habitats, but more likely to breed at or below treeline
	*Rare (R)*	Incidental; alpine breeder only under localized, specific habitat conditions
**Migration behaviour**		**General migration distance**
	*Resident (R)*	Resident year-round, allowing for occasional, temporary movements in response to extreme weather or social factors (i.e., multi-species flock)
	*Short (S)*	Seasonal altitudinal, nomadic, or short-distance latitudinal migrant ( < 3,000 km), but remains within general region
	*Long (L)*	Involves major latitudinal shift ( > 3,000 km) and change in region (i.e., temperate to tropics)
**Nest type**		**Predominant nest structure for alpine breeding birds**
	*Open*	Open cup nest
	*Cavity*	Nest placed in burrows, rock crevices, caves, or tree cavities
	*Domed*	Nest with a roof built by the bird; globular, spherical, or semi-domed
	*BP*	Brood parasite; lays eggs in any of the above nest types.
**Nest site**		**Typical nest placement or substrate**
	*Ground*	On the ground, under a shrub or grass clump, or next to a rock
	*Bank*	Cavity nest dug into the ground or a bank (i.e., a burrow/tunnel)
	*Shrub*	Placed above ground in vegetation (i.e., shrub, stunted tree, bamboo, sedge)
	*Tree*	Placed in branches or a cavity (natural, excavated) of a stunted tree
	*Cliff*	Cliff ledge or against a vertical wall (e.g., many raptor or swallow nests)
	*Rock*	Cavity nest placed within a natural rock cavity or crevice
	*Glacier*	Placed in a cave or crevice within a perennial ice field
**Data reliability**		**An uncertainty index based on described nest traits**
	*0*	Nest traits are undescribed
	1	Less than 5 nests have been described
	2	More than 5 nests described, but only from a single population
	3	Nests described from multiple populations and regions.
**Endemism**		**Endemic to a specific mountain region**
	*Species*	Species range is restricted by physical, environmental, or biological barriers to a mountainous region (e.g., endemic to Tasmania)
	*Subspecies*	Same as above for recognized subspecies
	*Non-endemic*	Monotypic; broad distribution across multiple regions
**IUCN status**		**Global conservation status according to the IUCN red list**

### Data uncertainty

Globally, there is significant variation in accessibility to alpine habitats and funding for alpine research. As a result, uncertainty in alpine breeding status may differ among regions and species. For example, in New Guinea, mist-net surveys and point counts across elevation have identified species that frequently use alpine habitat, but a dearth of breeding biology studies means that there are few nest records above treeline. It is thus necessary to codify this level of uncertainty for each species.

To this effect, we included a variable termed ‘Data reliability’, which is a four-level categorical variable from 0 to 3 that is based on the number of reported nests that have been found and described for each species. We used the presence of nest descriptions to evaluate uncertainty because active nests are the must fundamental form of evidence for breeding above treeline, and therefore it is reasonable that a species with less existing knowledge about nest traits or nesting behaviours will have considerably more uncertainty around its designation as an alpine breeding species. For this variable, 0 indicates that nest traits are undescribed for a given species, 1 means less than five nests have been described, 2 indicates more than five nests have been described, but all from a single population, and therefore there is limited understanding of geographic variation, while 3 occurs when nests have been described from multiple populations or regions. If nest traits were undescribed for a species (data reliability = 0), then nest type and site were inferred from the most closely related species with available data, and whenever possible, a congener was selected that also breeds at high elevations or in alpine habitats. While the nest traits of most species have been sufficiently described, there is a significant proportion of alpine breeding birds with less available data (27.0%; Fig. 3i). The relative number of described nests was derived from Birds of the World^30^. We recognize that these data may not reflect true knowledge of nest traits given that not all species accounts have been recently updated. However, it does represent a consistent data source that allowed us to approximate data reliability sufficiently for our purposes.

In combination, data reliability and alpine breeding status fully characterize alpine breeding uncertainty. For example, a species considered a rare alpine breeder with a data reliability of 3, means that there is strong evidence for breeding above treeline, but only incidentally under very specific circumstances. However, a rare alpine breeder with a data reliability of zero (i.e., nest undescribed), means that the likelihood of breeding above treeline may be probable based on behavioural observations, but further confirmation is required. When using this dataset for analyses, one must decide whether to use a conservative approach or consider all potential alpine breeding species with the appropriate caveats (see Usage Notes).

## Data Records

The global alpine breeding bird dataset can be accessed from the *Figshare* repository at 10.6084/m9.figshare.20556750^35^. The database is formatted as an Excel Workbook that includes two primary datasets under the tabs labelled ‘Global alpine breeding birds’ and ‘Regional alpine breeding birds’. For the **Global alpine breeding bird dataset**, each row corresponds to a single species. All species traits, including breeding specialization, alpine breeding status, migration behaviour, nest type, nest site, migration behaviour, and mountain endemism represent the predominant trait expression for alpine breeding populations across the major mountain regions. A species is an alpine specialist in this case only if it primarily breeds above treeline in all regions. For the **Regional alpine breeding bird dataset**, a species may be included in one to 12 rows according to the number of mountain regions it occurs in (Tables 2, 3). In this way, alpine breeding categories and species traits are specific to each mountain region for a particular species. For example, Bar-headed goose (*Anser indicus*) occupies three rows in the regional dataset, corresponding to North Asia, Tibet, and the Hindu Kush-Himalayan Arc, including information that this species has primarily been recorded nesting on the ground in the Himalayas, but on cliffs in the Altai Mountains. Note that certain variables, such as the IUCN status or Data reliability, are included in the global dataset only since these are based on all available data and are not region-specific.

In addition to the primary datasets, two additional tabs are included: ‘Metadata’ and ‘Data sources’. Metadata includes full descriptions of each variable, whether the variable applies to the global or regional dataset, any relevant notes to explain the choice of category levels, and the primary sources for the variable. Descriptions of each variable can also be found in Table 4, but those in the Metadata are expanded to provide additional detail. The Data sources tab includes the primary literature references used to determine alpine breeding status or treeline elevation organized by major region. Note that expert knowledge, technical reports, and bird trip reports or blogs are also stated generally within the data sources list, since they were fundamental to assembling the dataset, but cannot be sourced through traditional literature reviews. The column ‘Data type’ indicates whether the reference provided information on alpine breeding status or on treeline elevation, which helped us define the criteria for alpine breeding.

## Technical Validation

We followed a set of steps when gathering information for this dataset to ensure data quality control and a hierarchical internal review process. First, the regional co-authors and experts assembled a list of species with evidence of breeding above treeline. For each species, the lead author (DRD) conducted a literature search to independently confirm recorded evidence of breeding above treeline using published sources such as Birds of the World^30^, local breeding bird atlases, and scientific literature searches in the web search engines ‘Google Scholar’ and ‘Web of Science’ combining a species’ common or scientific name with the terms ‘alpine’, ‘breeding’, ‘nesting’, ‘high elevation’, and ‘above treeline’. If there were concerns about the inclusion of a species, DRD and the regional experts discussed available alpine breeding evidence and, when needed, solicited advice from additional sources such as local researchers or bird guides for observations of breeding behaviour (e.g., a nesting record, carrying nesting material, provisioning, or dependent offspring). In addition, DRD independently conducted a literature review for any species that occurred within a mountain region but was not included in the initial regional list. These species were derived from a global dataset of elevational ranges, which includes 8,685 species that occur within the boundaries surrounding the world’s mountainous regions^12^. If there was evidence or reasonable suspicion that a species would breed above treeline, these additional potential species were vetted by the regional co-authors, and again, advice was solicited from additional parties if needed. In total, approximately 400 species were confirmed as alpine breeders through expert solicitation. This hierarchical, stepwise approach of filtering species helped to maximize the available knowledge for each region.

The final alpine breeding bird list for each region was proofed and organized by DRD and then reviewed by the regional experts to eliminate any errors. All datasets from individual regions were combined into the final global and regional datasets and reviewed again by multiple authors. We note that a few species within each region probably were not identified as alpine breeders due to limited observations, leading to underestimation of the true global tally of alpine breeding birds. Overall, however, our approach should capture most alpine species and represents a step towards characterizing global alpine bird communities. The purpose of this dataset is to provide a global baseline of alpine breeding birds that can be refined in the future as additional data is collected. Updated versions of the database will be stored in the same online repository.

## Usage Notes

These data can be used to address large-scale ecology, evolution, and biogeography questions underlying global alpine bird communities. Use of these data when evaluating the ecology and conservation of alpine birds must operate with the understanding that species traits, such as nest type, nest site, or migration often vary among subspecies and populations given the latitudinal and longitudinal extremes over which alpine ecosystems occur. We therefore recommend carefully considering the spatial and temporal scale of any analysis stemming from this dataset and the reliability of inferring population-level patterns from predominantly species-level traits. To partly address this issue, we provided a regional dataset that includes within-species trait differences among the major global regions, allowing for certain finer-scale analyses. Importantly, we note that regions differ in their data reliability (see *Data uncertainty*). A species may be well known and documented as an alpine breeder in one region but may have limited or no observations in another region. In general, inaccessibility is a feature of high elevation habitats and alpine habitats often receive less research focus and funding in certain regions. We hope this dataset will be expanded and refined as new data become available.

## Data Availability

No code was used to generate this dataset.
